# Supplementary material for: Genomic Diversity and Phenotypic Variation in Fungal Decomposers Involved in Bioremediation of Persistent Organic Pollutants
Source: J Fungi (Basel). 2023 Mar 29;9(4):418. doi: 10.3390/jof9040418 (PMC10145412; doi:10.3390/jof9040418)
Supplement: Supplementary file 1 [file jof-09-00418-s001.zip › fungal supplememntary figures.pptx]

## Slide 1
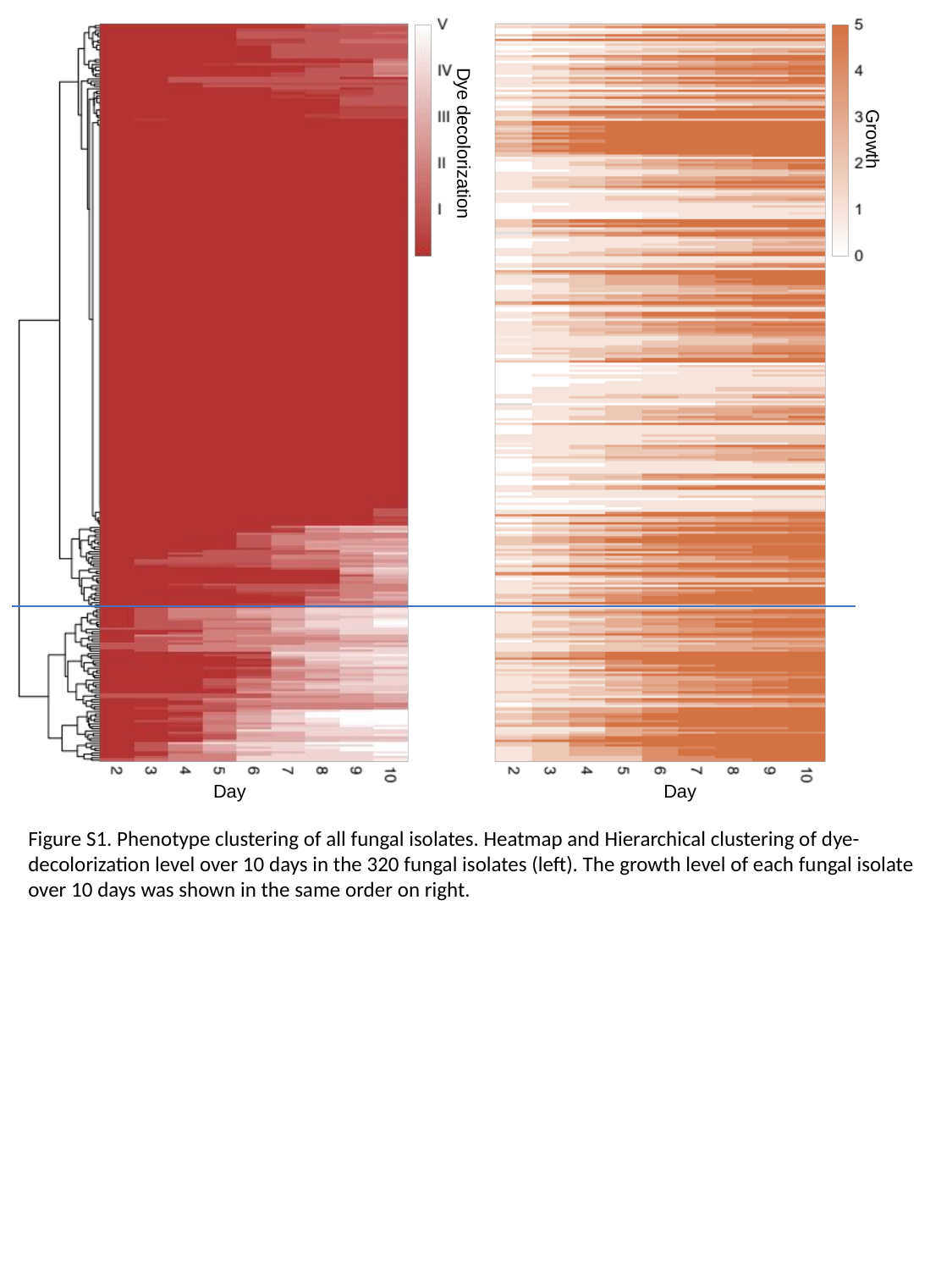

Growth
Dye decolorization
Day
Day
Figure S1. Phenotype clustering of all fungal isolates. Heatmap and Hierarchical clustering of dye-decolorization level over 10 days in the 320 fungal isolates (left). The growth level of each fungal isolate over 10 days was shown in the same order on right.

## Slide 2
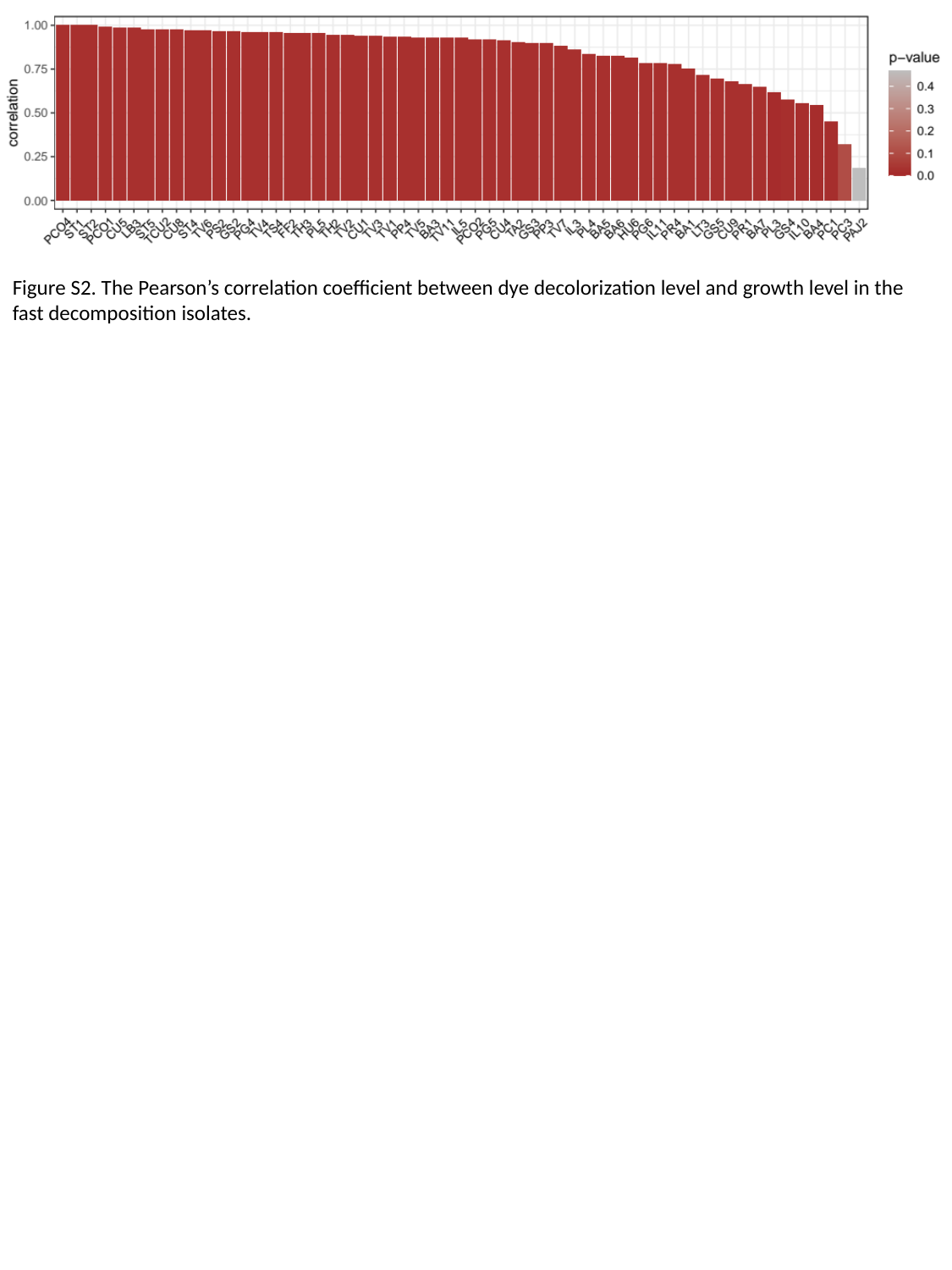

Figure S2. The Pearson’s correlation coefficient between dye decolorization level and growth level in the fast decomposition isolates.
